# The association between levels of inflammatory markers in autistic children compared to their unaffected siblings and unrelated healthy controls

**DOI:** 10.3906/sag-1812-167

**Published:** 2019-08-08

**Authors:** Loai ALZGHOUL*, Sultan S. ABDELHAMID, Ahmad H. YANIS, Yasmeen Z. QWAIDER, Mohammad ALDAHABI, Suzan A. ALBDOUR

**Affiliations:** 1 Department of Physiology and Biochemistry, The University of Jordan, Amman Jordan; 2 School of Medicine, The University of Jordan, Amman Jordan

**Keywords:** Autism, interleukins, siblings, cytokines, Jordan

## Abstract

**Background/aim:**

Autism spectrum disorder (ASD) describes a range of neurodevelopmental disabilities that impair behavior and communication. Although it is relatively prevalent, the pathophysiology is still subject to speculation and debate. The aim of this study is to identify a possible association between interleukin-6, -8, -9, and -10 and tumor necrosis factor alpha (TNF-α) in autism among Jordanian children by comparing the plasma levels of these cytokines in autistic children with those of their unaffected siblings and unrelated healthy controls.

**Materials and methods:**

In this study, 80 Jordanian children under the age of 12 with diagnosed autism were selected. For comparison, 51 unaffected siblings and 86 unrelated healthy controls were also recruited. Venous blood was collected and interleukin levels in all three groups were investigated.

**Results:**

Interleukin-6 was found to be significantly higher in the plasma of both autistic children and their siblings compared with the unrelated healthy control group (P < 0.05). As for interleukin-8 and TNF-α, plasma levels were significantly higher exclusively in autistic children compared to their siblings and unaffected control subjects (P < 0.001, P < 0.001). There was no significant difference between plasma levels of the previously mentioned cytokines in the siblings and the unrelated control group. As for interleukin-9 and interleukin-10, no significant differences were found between all three groups (P = 0.15, P = 0.35).

**Conclusion:**

We found that interleukin-8 and TNF-α were exclusively elevated in autistic Jordanian children, while interleukin-6 was elevated in both autistic children and their siblings, potentially dismissing its significance. These results may lead to a better understanding of the disorder’s pathophysiology, early testing, and diagnosis.

## 1. Introduction

Autism spectrum disorder (ASD) is a heterogeneous disorder that consists of a range of neurodevelopmental disabilities causing significant behavioral and communicational deficits [1]. According to the Centers for Disease Control and Prevention, about 1 in 59 children aged 8 years old are diagnosed with ASD in the United States with a male predisposition [2]. Studies among Arabs and in the Middle East are few, but a recent study in Lebanon revealed a comparable prevalence, which was 1 in 66 among toddlers 16–48 months old [3]. While there are no official statistics about the prevalence of autism in Jordan due to the lack of local comprehensive studies in this field, experts affirm that there are about 8000 autistic individuals. Although the prevalence of ASD is significant, the etiology, which constitutes an interaction between genetics and environment, is largely unknown.

Recently, several studies have suggested a correlation between ASD and abnormal immune activation [4]. For instance, there is a high incidence of autistic subjects in families with other autoimmune disorders such as celiac disease and rheumatoid arthritis, suggesting a potential role for immune dysfunction in the pathophysiology of ASD [5]. At the cellular level, studies revealed skewed and dysfunctional immune cell populations in children with ASD. Moreover, children with ASD have an increased total number of peripheral blood monocytes, and their monocytes produce significantly higher amounts of cytokines in response to stimulation [6,7]. 

Cytokines are a superfamily of proteins that are an integral part of the signaling network between cells. In addition to being the primary regulators of inflammation and coordinating the response to infection and associated immune challenges, cytokines are involved in many physiological processes including growth, development, and cell differentiation as well. It was previously thought that the peripheral immune system does not cross the blood–brain barrier (BBB), but overwhelming new evidence suggests that it might play a role in neuronal functions. For instance, several proinflammatory cytokines can cross the BBB, affect the hypothalamus, and trigger so-called sickness behavior [8]. Cytokines can also modulate brain function, affecting cognitive and emotional processing, and might be responsible for the mood and sleep disturbances commonly seen in children with ASD [9]. A number of recent studies have demonstrated an increase in proinflammatory cytokines and a decrease in antiinflammatory cytokine levels in children with ASD compared with healthy individuals [10]. Not all cytokines were associated with ASD; among the most replicated results were higher levels of interleukin-6 (IL-6), interleukin-8 (IL-8), and tumor necrosis factor-α (TNF-α), and lower levels of the antiinflammatory interleukin-10 (IL-10) [11–13]. On the other hand, interleukin-9 (IL-9) is a proinflammatory cytokine that modulates the development of ion channels and action potentials in cultured brain neurons. However, several studies revealed no association between IL-9 blood levels and the pathophysiology of ASD. 

ASD has always been regarded as a predominantly genetic disorder. A recent twin-study metaanalysis demonstrated a heritability estimate of 64%–91% [14]. However, other potential contributing factors in the pathophysiology of ASD can be environmental or genetic/environmental interactions. A previous study has shown a slight decrease in B-cell levels in children with ASD as well as their unaffected siblings [15], while other studies found that both children with ASD and their unaffected siblings have antibrain antibodies in their blood [16]. Hence, investigating environmental and immunological factors between children with ASD and their unaffected siblings can reduce heterogeneity and assist in the identification of immunological factors involved with ASD. Therefore, the aim of this study is to investigate the plasma levels of IL-6, IL-8, IL-9, IL-10, and TNF-α in autistic children compared with their unaffected siblings and unrelated healthy control children. 

## 2. Materials and methods

### 2.1. Sample 

A total of 96 autistic Jordanian children (81 males, 19 females) younger than 12 years old diagnosed with ASD by experienced child psychiatrists and neuropediatricians according to the Diagnostic and Statistical Manual of Mental Disorders (fifth edition) were initially recruited for this study from pediatric clinics and local special healthcare centers, along with their siblings. After written consent from their parents was obtained, the ASD group was interviewed by a team of child psychiatrists, as well as the principal investigator, to obtain the medical history and achieve a unified diagnosis. They fell within the subclass of “Childhood Autism” according to Section F84.0 in the 10th revision of the International Statistical Classification. Control subjects were recruited from among visitors to our local university hospital for any reason other than acute or chronic disease, and/or children who accompanied a family member to the hospital. This study was approved by the research committee of the School of Medicine at our institute and the ethics committee of our institute’s hospital (reference number 10/2018/1181), and it was carried out in accordance with ethical standards.

Karyotyping was performed for all autistic subjects to exclude syndromic autism and genetic abnormalities that are associated with ASD, resulting in the exclusion of two male subjects. Other exclusion criteria included chronic illnesses, allergies, or symptoms of illnesses and infections currently or in the last 3 months and a restricted diet or supplementation regimen. As a result, a total of 16 subjects (14 males and 13 females) were excluded. Fifty-one siblings of the autistic children and 86 unrelated healthy controls (UHCs) who did not have a family history of autism were matched for sex and age and assigned as the sibling and UHC groups, respectively. Descriptive data can be found in the Table.

**Table:** Descriptive table of study subjects.

Group	Number	Mean age	SD
Autistic subjects			
Female	13	5.87	1.80
Male	67	6.60	2.80
Total	80	6.48	2.66
Siblings			
Female	19	6.70	2.00
Male	32	6.26	2.71
Total	51	6.43	2.45
Controls			
Female	45	6.35	2.54
Male	41	5.81	2.52
Total	86	6.10	2.53

### 2.2. Blood collection and interleukin measurement

A total of 3 mL of fasting venous blood was collected from each participant into a potassium EDTA tube. Following centrifugation, the plasma was transferred to an Eppendorf tube and stored at –80 °C to be used for quantification of interleukin levels. Plasma interleukin levels were measured using specific individual ELISA kits for each of IL-6, IL-8, IL-9, IL-10, and TNF-α (RayBiotech Inc., Norcross, GA, USA) following the manufacturer’s recommendations. ELISA plates were read by a BioTek plate reader (Winooski, VT, USA), and a graph of interleukin levels was plotted using the reader’s software (Gen5). All samples were measured three times each. Samples with spurious measurements were removed from the study (six samples: four from the ASD group and two from the control group). Data were then averaged for each sample and run for statistical analysis. 

### 2.3. Statistical analysis 

For quantitative differences in plasma interleukin levels between groups, statistical differences were investigated using one-way analysis of variance (ANOVA) with Bonferroni post hoc pairwise comparisons. When sex was found as a confounding factor that could influence interleukin differences between groups, linear regression analysis was used to adjust for sex. All statistics were evaluated using SPSS and P < 0.05 was considered to be significant.

## 3. Results

The levels of IL-6, IL-8, IL-9, IL-10, and TNF-α were measured in autistic children and compared to the levels of their unaffected siblings and UHCs. We also tested for differences between age and the interleukins using Pearson’s correlation coefficient, yielding no significant results. 

**Figure 1:**
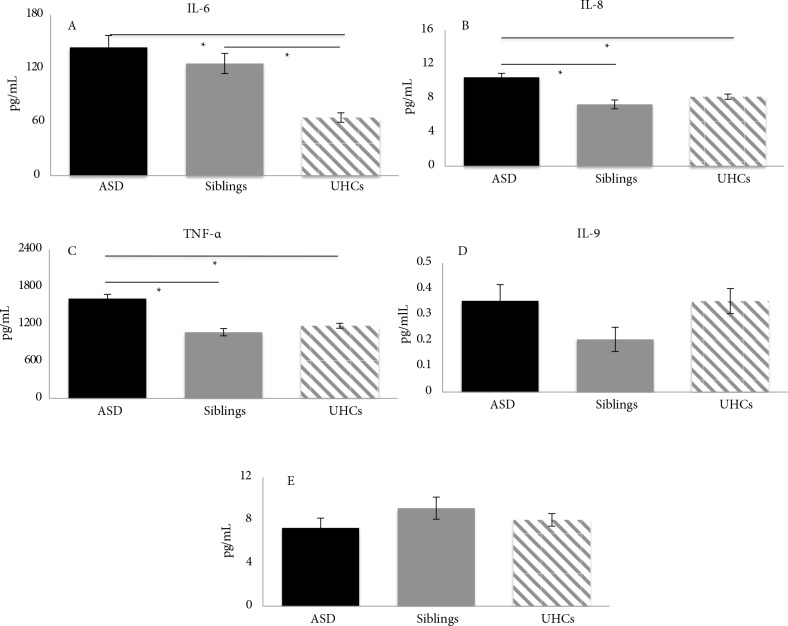
Mean serum concentrations of cytokines amongst groups. The bar charts show the differences in mean serum concentrations of specific cytokines between ASD patients, siblings, and UHCs. A) Significantly higher IL-6 mean serum concentrations in ASD and siblings compared to UHCs but no significant difference between ASD and siblings. B, C)Mean serum concentrations of IL-8 and TNF-α, respectively, both showing significantly higher mean serum concentrations in ASD patients compared with UHCs and siblings but no significant difference between siblings and UHCs. D, E)Mean serum concentrations of IL-9 and IL-10, respectively, showing no significant difference in mean serum concentrations between any of the study groups. * P < 0.001.

One-way ANOVA for IL-6 revealed a statistical difference in plasma levels between the groups [F(2, 215) = 18.25, P < 0.001]. Pairwise comparison revealed a significantly higher level of IL-6 in both autistic subjects (M = 143.51, SD = 118.28) and their siblings (M = 125.54, SD = 80.89) compared to UHCs (M = 64.95, SD = 48.67) (P < 0.001). No significant difference was detected between the ASD group and siblings (P = 0.754), as shown in Figure 1A. 

For IL-8 plasma levels, one-way ANOVA revealed concentration differences between the groups [F(2, 215) = 14.22, P < 0.001]. Pairwise comparison revealed significantly higher plasma levels in the autistic group (M = 10.45, SD = 4.20) compared with both the siblings (M = 7.27, SD = 3.78) (P < 0.001) and UHCs (M = 8.16, SD = 2.85) (P < 0.001). However, pairwise comparisons did not show any significant differences in IL-8 plasma levels between the sibling and UHC groups (P = 0.488), as shown in Figure 1B. 

For TNF-α plasma levels, one-way ANOVA revealed concentration differences between the groups [F (2, 215) = 26.54, P < 0.001]. Pairwise comparison revealed significantly higher plasma levels in the autistic group (M = 1611, SD = 585.4) compared with both siblings (M = 1066, SD = 426.9) (P < 0.001) and UHCs (M = 1171, SD = 377.8) (P < 0.001). However, pairwise comparison did not show any significant difference in TNF-α plasma levels between the sibling and UHC groups (P = 0.633), as shown in Figure 1C. 

No significant differences between the tested groups were detected in IL-9 plasma levels [F(2, 215) = 1.94, P = 0.15] (P = 0.146) or IL-10 levels [F(2, 215) = 1.06, P = 0.35] (P = 0.348), as shown in Figures 1D and 1E.

## 4. Discussion

In this study, the analysis of plasma cytokine levels demonstrated differences between autistic Jordanian children, their healthy unaffected siblings, and a control group. Our data revealed significantly high levels of IL-6, IL-8, and TNF-α compared to those of UHCs, but no significant difference was observed for IL-9 and IL-10 levels. When cytokine levels were compared between autistic children and their healthy siblings, significantly higher levels of IL-8 and TNF-α were found in the autistic group, while other cytokine analyses yielded no statistical difference. On the other hand, plasma IL-6 levels were higher in healthy siblings compared to UHCs. Given these results, we suggest that the immune dysfunction and the activation of immune responses involving the production of IL-8 and TNF-α may possibly have a more significant role in the pathophysiology of autism, while other immunological factors such as IL-9 have a less direct role in ASD development. Contrary to the literature, we believe that IL-6 has less impact on ASD development, as healthy unrelated siblings had high levels too. Immunological factors that involve IL-6 activation may only have a familial predisposition for ASD with minimal direct pathophysiological involvement. 

Our data have revealed elevated plasma levels of TNF-α in patients with ASD compared with both UHCs and siblings, suggesting a potential more direct role of TNF-α in the pathophysiology of ASD. Tumor necrosis factor-α is a proinflammatory cytokine that is a significant contributor to homeostatic function and pathophysiological processes in the central nervous system [17]. Pathological levels of TNF-α potentiate the neurotoxic effect of glutamate, leading to a synergistic induction of neuronal cell death [18]. This affects the normal embryonic development of the brain and also leads to the destruction of dendrites and synaptic connections [19]. A study showed that there were elevated levels of TNF-α in the amniotic fluid of mothers carrying children with ASD compared to a control group [20]. Another study demonstrated a correlation between higher TNF-α levels and the severity of ASD symptoms [21]. Likewise, Tsilioni et al. showed that children with ASD had significantly higher serum TNF-α levels compared to a control group. Reduction in serum TNF-α via a luteolin-containing diet was associated with improved behavioral outcomes in children with ASD [22]. This emphasizes the role of TNF-α in the pathophysiology of ASD. On the other hand, only a few studies showed that there were no significant differences or even lower levels of TNF-α in children with ASD compared to controls [23,24]. 

Similar to TNF-α, IL-8 was elevated specifically in the plasma of children with ASD. It is produced by phagocytic cells exposed to certain stimuli also produced by TNF-α [25], which might explain why IL-8 and TNF-α levels were mutually elevated in ASD subjects. Also, higher levels of IL-8 were associated with more aberrant behaviors in patients with ASD, including stereotypic behavior, hyperactivity, less linguistic abilities, and cognitive ability [26]. 

Another cytokine, IL-1β, is found to increase the production of IL-17, which in part is a potent mediator of IL-8 [27]. It is worth noting that both IL-1β and IL-17 are commonly elevated in patients with ASD. Once activated, IL-8 acts as a chemoattractant that attracts neutrophils to areas of inflammation, causing the release of proteolytic substances [28]. Therefore, elevated levels of IL-8 in both brain and CSF samples of individuals with ASD support a stronger role for IL-8 in the pathophysiology of ASD [29,30]. This study has revealed a significantly higher level of IL-8 in autistic children compared to their siblings. However, a similar study found no significant difference between autistic children and their siblings [31]. This inconsistency of results could be due to the different genetic backgrounds of the samples in the two groups, or the small sample size in the mentioned study.

As for IL-6, elevation of this cytokine has been a repeated finding in autistic children. In comparison, we found higher IL-6 levels in both children with ASD and their siblings compared with UHCs. More importantly, we found no significant plasma IL-6 level difference in children with ASD compared to their siblings. This finding is consistent with another study that found similar results [31]. Although it was previously classified as a proinflammatory cytokine and a major inducer of immune and inflammatory response [32], recent studies have shown an antiinflammatory role for IL-6 that prevents increased tissue damage when transitioning from innate to acquired immunity [33]. Furthermore, several studies showed that IL-6 can also be produced by nonimmune mediated cells such as mesenchymal cells, endothelial cells, and fibroblasts, among others, in response to various stimuli [34]. Some studies have revealed an increase in IL-6 levels after exercise without muscle damage [35]. Even in brain tissue, IL-6 can be expressed by certain astrocytoma and glioma lines under certain stimulation [36]. These data might indicate that IL-6 can be elevated in a wide range of conditions not limited to inflammation. Hence, its direct role in the pathophysiology of autism is questionable, which might also explain the absence of significance noticed in our results between children with ASD and their siblings. According to our results, since IL-6 is found in the unaffected healthy siblings, we believe its role in ASD pathophysiology might be overestimated. However, further in-depth study of its exclusive role in ASD development is necessary. 

We found no significant difference in IL-9 and IL-10 plasma levels between autistic children, siblings, or the control subjects. IL-9 also plays a vital role in autoimmune activation of the CNS, yet our results are in concordance with the literature. As for IL-10, Molloy et al., among others, found results similar to our study where IL-10 levels in peripheral blood mononuclear cells did not differ between ASD patients and control subjects despite the elevation of levels of other cytokines [37]. These results warrant more investigation of the possible roles of IL-9 and IL-10 in the pathophysiology of ASD.

This study has several limitations. Although our sample size for children with ASD is larger than others in the literature, we could not include all siblings and the analysis would be stronger with a more comprehensive siblings group. Also, all our ASD patients fell within one subtype, and including other subtypes would help to clarify things better. We hope to perform follow-up and check the interleukin levels for the same sample population further down the line, and we urge investigators to do prospective studies as well. 

In conclusion, our study was able to successfully highlight IL-8 and TNF-α as biomarkers potentially higher only in autistic children compared to other groups. Although not dismissible, IL-6 is high in both children with ASD and their healthy siblings in our sample, questioning its importance as a biomarker. We hope our results will help to better understand the disorder in terms of its clinical and pathophysiological aspects.

## Acknowledgment

This study was supported by the Deanship of Academic Research, The University of Jordan, Amman, Jordan.

## References

[ref0] (2016). One autism, several autisms. Phenotypical variability in autism spectrum disorders. Revista de Neurología.

[ref1] (2018). Prevalence of autism spectrum disorder among children aged 8 years. Surveillance Summaries.

[ref2] (2016). Prevalence of autism spectrum disorder in nurseries in Lebanon: a cross sectional study. Journal of Autism and Developmental Disorders.

[ref3] (2015). The impact of neuroimmune alterations in autism spectrum disorder. Frontiers in Psychiatry.

[ref4] (2009). Association of family history of autoimmune diseases and autism spectrum disorders. Pediatrics.

[ref5] (2003). High blood monocyte counts and neopterin levels in children with autistic disorder. American Journal of Psychiatry.

[ref6] (2010). Differential monocyte responses to TLR ligands in children with autism spectrum disorders. Brain, Behavior, and Immunity.

[ref7] (2004). Cytokine-induced sickness behaviour: a neuroimmune response to activation of innate immunity. European Journal of Pharmacology.

[ref8] (2006). The immune response in autism: a new frontier for autism research. Journal of Leukocyte Biology.

[ref9] (2017). The role of the immune system in autism spectrum disorder. Neuropsychopharmacology.

[ref10] (2015). Inflammatory cytokines: potential biomarkers of immunologic dysfunction in autism spectrum disorders. Mediators of Inflammation.

[ref11] (2011). Elevated plasma cytokines in autism spectrum disorders provide evidence of immune dysfunction and are associated with impaired behavioral outcome. Brain, Behavior, and Immunity.

[ref12] (2013). Targeting T-helper 9 cells and interleukin-9 in autoimmune diseases. Cytokine & Growth Factor Reviews.

[ref13] (2016). Heritability of autism spectrum disorders: a meta-analysis of twin studies. Journal of Child Psychology and Psychiatry.

[ref14] (2009). An autistic endophenotype results in complex immune dysfunction in healthy siblings of autistic children. Biological Psychiatry.

[ref15] (2006). Antibrain antibodies in children with autism and their unaffected siblings. Journal of Neuroimmunology.

[ref16] (2014). Tumor necrosis factor alpha: a link between neuroinflammation and excitotoxicity. Mediators of Inflammation.

[ref17] (2005). TNFα potentiates glutamate neurotoxicity by inhibiting glutamate uptake in organotypic brain slice cultures: neuroprotection by NFκB inhibition. Brain Research.

[ref18] (1994). Tumor necrosis factor-alpha potentiates glutamate neurotoxicity in human fetal brain cell cultures. Developmental Neuroscience.

[ref19] (2013). Amniotic fluid inflammatory cytokines: potential markers of immunologic dysfunction in autism spectrum disorders. World Journal of Biological Psychiatry.

[ref20] (2017). Immunological cytokine profiling identifies TNF as a key molecule dysregulated in autistic children. Oncotarget.

[ref21] (2015). Children with autism spectrum disorders, who improved with a luteolin-containing dietary formulation, show reduced serum levels of TNF and IL-6. Translational Psychiatry.

[ref22] (2015). Inflammatory activity in autism spectrum disorder. Advances in Experimental Medicine and Biology.

[ref23] (2014). /glutamatergic imbalance relative to excessive neuroinflammation in autism spectrum disorders. Journal of Neuroinflammation.

[ref24] (1992). Interleukin-8, a chemotactic and inflammatory cytokine. FEBS Letters.

[ref25] (2011). Elevated plasma cytokines in autism spectrum disorders provide evidence of immune dysfunction and are associated with impaired behavioral outcome. Brain, Behavior, and Immunity.

[ref26] (2010). IL-1β-mediated signals preferentially drive conversion of regulatory T cells but not conventional T cells into IL-17-producing cells. Journal of Immunology.

[ref27] (1993). The role of interleukin-8 in inflammation and mechanisms of regulation. Journal of Periodontology.

[ref28] (2005). Neuroglial activation and neuroinflammation in the brain of patients with autism. Annals of Neurology.

[ref29] (2009). Elevated immune response in the brain of autistic patients. Journal of Neuroimmunology.

[ref30] (2013). Plasma cytokine profiling in sibling pairs discordant for autism spectrum disorder. Journal of Neuroinflammation.

[ref31] (2006). Interleukin-6 biology is coordinated by membrane-bound and soluble receptors: role in inflammation and cancer. Journal of Leukocyte Biology.

[ref32] (2011). The pro- and anti-inflammatory properties of the cytokine interleukin-6. Cell Research.

[ref33] (1989). 6 inhibits lipopolysaccharide-induced tumor necrosis factor production in cultured human monocytes, U937 cells, and in mice. Journal of Immunology.

[ref34] (2006). The role of il-6 in mediating the anti-inflammatory effects of exercise. Journal of Physiology and Pharmacology.

[ref35] (2012). Interleukin-6, a major cytokine in the central nervous system. International Journal of Biological Sciences.

